# Zirconium Oxynitride Thin Films for Photoelectrochemical Water Splitting

**DOI:** 10.1021/acsaem.4c00303

**Published:** 2024-04-27

**Authors:** Verena Streibel, Johanna L. Schönecker, Laura I. Wagner, Elise Sirotti, Frans Munnik, Matthias Kuhl, Chang-Ming Jiang, Johanna Eichhorn, Saswati Santra, Ian D. Sharp

**Affiliations:** † Walter Schottky Institute, Technical University of Munich, Garching 85748, Germany; ‡ Physics Department, TUM School of Natural Sciences, Technical University of Munich, Garching 85748, Germany; § Institute of Ion Beam Physics and Materials Research, Helmholtz-Zentrum Dresden-Rossendorf (HZDR), Dresden 01328, Germany

**Keywords:** zirconium oxynitride, reactive sputtering, thin film photoanodes, photoelectrochemical water splitting, oxygen evolution reaction, water oxidation

## Abstract

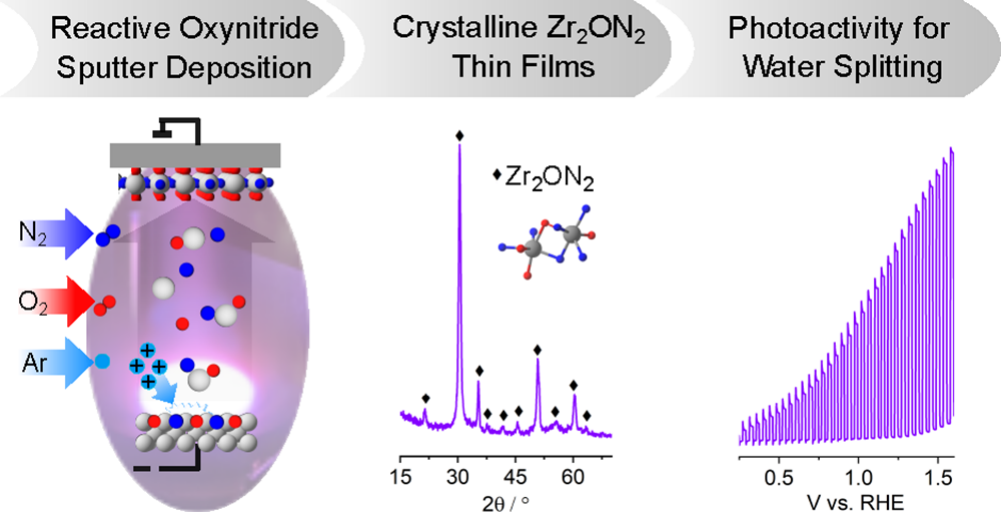

Transition metal oxynitrides are a promising class of functional materials for photoelectrochemical (PEC) applications. Although these compounds are most commonly synthesized via ammonolysis of oxide precursors, such synthetic routes often lead to poorly controlled oxygen-to-nitrogen anion ratios, and the harsh nitridation conditions are incompatible with many substrates, including transparent conductive oxides. Here, we report direct reactive sputter deposition of a family of zirconium oxynitride thin films and the comprehensive characterization of their tunable structural, optical, and functional PEC properties. Systematic increases of the oxygen content in the reactive sputter gas mixture enable access to different crystalline structures within the zirconium oxynitride family. Increasing oxygen contents lead to a transition from metallic to semiconducting to insulating phases. In particular, crystalline Zr_2_ON_2_-like films have band gaps in the UV–visible range and are n-type semiconductors. These properties, together with a valence band maximum position located favorably relative to the water oxidation potential, make them viable photoanode candidates. Using chopped linear sweep voltammetry, we indeed confirm that our Zr_2_ON_2_ films are PEC-active for the oxygen evolution reaction in alkaline electrolytes. We further show that high-vacuum annealing boosts their PEC performance characteristics. Although the observed photocurrents are low compared to state-of-the-art photoanodes, these dense and planar thin films can offer a valuable platform for studying oxynitride photoelectrodes, as well as for future nanostructuring, band gap engineering, and defect engineering efforts.

## Introduction

Transition metal (TM) oxynitrides are an emerging class of functional materials with electronic and optical properties that can be controllably tuned by variation of both cation and anion compositions.^1,2^ Although oxynitrides are generally less thermodynamically stable than their respective oxides,^3^ the additional compositional and structural degrees of freedom enable modification of their energetic band gaps, valence/conduction band positions, and surface chemistries. Furthermore, nitrogen increases bond covalency compared to oxygen, which may improve charge transport and reduce electron–hole recombination in semiconducting oxynitride phases.^4^ Thus, TM oxynitride compounds offer considerable promise as semiconductor photoelectrodes for photoelectrochemical (PEC) energy conversion applications, with prospects for enhanced stability relative to nitrides and improved visible light absorption and charge transport compared to oxides. However, oxynitrides can be challenging to synthesize because of the high stability of molecular nitrogen and the lower electronegativity of nitrogen compared to oxygen.^5^ For these reasons, oxynitrides are usually fabricated by high-temperature ammonolysis of metals or metal oxides.^6^ Unfortunately, these harsh synthesis conditions are energy-intensive and can be incompatible with commonly used substrates. For example, transparent conductive oxides (TCOs) that are typically utilized as back contacts in tandem absorber systems are not stable under the ammonia annealing conditions used for synthesis of oxynitride compounds.^7^ Hence, although a wealth of literature exists on particle-based oxynitride photocatalysts for overall water splitting,^2,8−10^ this class of materials remains underexplored for scalable thin film-based solar fuel generators.

Zirconium oxynitrides represent a promising material system within the oxynitride space. These compounds are already utilized for a broad range of applications, including as wear- and corrosion-resistant coatings,^11^ oxygen reduction reaction^12^ and ammonia decomposition catalysts,^13^ gate dielectrics,^14^ and temperature sensors.^15^ Compared to tantalum oxynitride, which is perhaps the most intensively investigated oxynitride compound for photoelectrochemical energy conversion,^16^ zirconium is more abundant and more readily available.^17^ In addition, theoretical studies predict that zirconium oxynitrides have suitable energy band alignment to function as photoanodes.^18−20^ Nevertheless, to the best of our knowledge, there are so far no experimental investigations testing these theoretical predictions for thin-film photoanodes. However, for particle-based systems, the photocatalytic activity of Zr_2_ON_2_ using sacrificial agents has been reported,^21^ and it is known that doping of tantalum oxynitride with zirconium can lead to an approximately 1 order of magnitude increased overall water-splitting activity.^22^

Herein, we use reactive sputter deposition to grow zirconium oxynitride thin films with variable oxygen-to-nitrogen ratios. This plasma-based nonequilibrium deposition approach allows the synthesis of oxynitride films at significantly milder processing conditions and with more precise control over composition than traditional high-temperature ammonolysis treatments.^23^ Comprehensive structural, compositional, and optical characterization reveals that the anion composition (O/N ratio) can be tuned over a broad range, enabling synthetic access to the metallic nitride, semiconducting oxynitride, and insulating oxide phases. At intermediate O/N ratios, polycrystalline films of semiconducting Zr_2_ON_2_ exhibiting nondegenerate n-type conductivity and band gaps within the UV–visible range can be formed. Combining ultraviolet photoelectron spectroscopy and UV–vis spectroscopy, we construct an energy band diagram that confirms that the Zr_2_ON_2_ band edges straddle the water oxidation and reduction potentials. Using chopped linear sweep voltammetry, we show that these films function as active photoanodes. Finally, we demonstrate that this photoactivity can be enhanced through vacuum annealing, which results in oxygen enrichment of the surface and elimination of dark-conductive channels present in the as-grown films.

## Experimental Section

### Thin Film Deposition

Zr_*x*_O_*y*_N_*z*_ thin films were deposited with a PRO Line PVD 75 Sputter Deposition System from Kurt J. Lesker Company. The thin films were deposited simultaneously on double-side polished fused silica substrates (Siegert Wafer GmbH) and n^+^-doped Si(100) wafers (<0.005 Ω·cm, Siegert Wafer GmbH). Before film deposition, silica substrates were cleaned in 1 vol % Hellmanex solution for 20 min in an ultrasonic bath, rinsed with water, sonicated again in acetone for 10 min, rinsed with isopropanol, and finally dried under dry N_2_ flow. The Si wafers were cleaned using the same procedure, excluding the initial Hellmanex step. Both substrate types were mounted centrally on a substrate holder that was transferred into the sputter deposition chamber via a load lock and rotated at 10 rpm during heating and deposition. To reach the deposition temperature of 600 °C, the substrates were heated in vacuum by an infrared lamp with a ramp rate of 10 °C/min. The base pressure of the sputter chamber was below 10^–7^ mbar and remained below 10^–6^ mbar after heating the substrate to 600 °C. The distance between the 2” diameter Zr target (Kurt J. Lesker, Zr702) and the substrate was approximately 20 cm (10 cm for some samples, as noted).

Target conditioning was performed after heating the substrate. For this purpose, the Zr target was initially sputtered for 15 min in pure Ar atmosphere (40 sccm, 1.3 × 10^–2^ mbar, Linde Electronics GmbH, 99.9999%) at 60 W DC power with the substrate shutter closed. During the last 5 min of this target conditioning process, an additional 20 W radio frequency (RF) bias was applied to the substrate holder to sputter-clean the substrate surfaces, particularly to remove the native oxide layer on the Si wafers. Thereafter, the Zr target was further conditioned in the reactive process gas mixture, which consisted of 10 sccm Ar, 20 sccm N_2_ (Linde Electronics GmbH, 99.9999%), and varying amounts of O_2_ (0–0.32 sccm, Linde Electronics GmbH, 99.9999%) at a total pressure of ∼9.5 × 10^–3^ mbar. During this step, the sputter power remained at an average power of 60 W but was switched to pulsed DC mode with 100 kHz repetition rate and 90% duty cycle. After the target potential reached steady state (usually after 10–30 min), the substrate shutter was opened, and the deposition was initiated. Films were deposited for 90 min, after which the samples were cooled down to 25–50 °C in vacuum, extracted from the sputter system through the ambient atmosphere, and then stored in an N_2_ desiccator.

### Structural, Compositional, and Optical Characterization

A Rigaku SmartLab X-ray diffractometer was used for structural characterization of the deposited thin films. Measuring in grazing incidence X-ray diffraction (GIXRD) geometry (0.4 or 0.5° incident angle) with Cu Kα radiation, the 2θ diffraction angle was scanned from 15 to 70° with a step size of 0.02° at a rate of 2° min^–1^. The same instrument was used to measure X-ray reflectivity (XRR), in which the 2θ/ω angle was scanned between 0 and 4° with a 0.01° step size during the course of 15 min runs. The XRR data were fitted with the SmartLab software to obtain approximate film thicknesses. XRD references were generated by retrieving crystal structures from the Crystallography Open Database (COD)^24^ or Materials Project^25^ and calculating the reference patterns using VESTA.^26^

The film compositions were assessed using energy-dispersive X-ray spectroscopy (EDX) in a Zeiss EVO SEM equipped with a Quantax (Bruker) X-ray detector. Measurements were performed using an incident electron energy of 10 keV and a working distance of 10 mm. To account for inhomogeneities, three different spots were measured and averaged for each sample, yielding variations of less than ±5%.

For selected samples, elastic recoil detection analysis (ERDA), including Rutherford scattering of the incoming ions, was used for depth-resolved quantification of the composition. These measurements were collected at the Helmholtz-Zentrum Dresden-Rossendorf (HZDR). For ERDA, a 43 MeV Cl^7+^ ion beam at an angle of 75° between sample normal and incoming beam, a 30° scattering angle, and an analysis area of approximately 2 × 2 mm^2^ were used. Recoil and scattered ions were detected in a Bragg ionization chamber. The analysis of the measurements was performed with the program NDF v9.3 g.^27^

The surface composition and electronic structure of the films were characterized via X-ray photoelectron spectroscopy (XPS) using a home-built system equipped with a SPECS Phoibos 100 analyzer and a nonmonochromatized Al Kα X-ray source (1486.6 eV). The recorded spectra were aligned to the adventitious carbon signal (284.8 eV), analyzed, and fitted with CasaXPS.

Ultraviolet photoelectron spectroscopy (UPS) was measured using the 21.2 eV He I line from a commercial He gas discharge lamp (Focus HIS 13). The photoemission spectra were collected using a SPECS Phoibos 150 hemispherical analyzer with a pass energy of 5 eV. A voltage of −10 V was applied to the sample to measure the secondary electron cutoff. Shown spectra have been shifted to account for the applied bias and reflect the actual electron binding energy.

The film morphology was probed by atomic force microscopy (AFM) using a Bruker Dimension Icon under ambient conditions and scanning electron microscopy (SEM) using an NVision 40 (Zeiss) under vacuum conditions. For all AFM measurements, PeakForce Mode and cantilevers with a nominal spring constant of 3 N m^–1^ (RFESPA-75, Bruker) were used.

Optical properties of thin film samples were characterized using variable angle spectroscopic ellipsometry and transmission measurements with a J.A. Woollam M-2000X ellipsometer. The ellipsometric parameters Psi and Delta were measured between 211 and 1688 nm at reflection angles between 45 and 75°. The obtained data were analyzed using the CompleteEASE software. The fit routine consisted of first applying a purely mathematical B-spline fit that served as the basis for a general oscillator (GenOsc) model of the deposited layer. For this model, fitting was constrained to conform to Kramers–Kronig consistency, with Cody–Lorentz and Tauc–Lorentz oscillators used to model band-to-band transitions, along with a Drude model for free carrier absorption.^28^

Contact potential difference (CPD) measurements with a KP020 setup by KP Technology, equipped with a gold tip, were used to determine the sample work function (Φ_sample_). Before the actual measurement, a calibration measurement using a freshly cleaved HOPG (highly oriented pyrolytic graphite) reference (Φ_ref_ = 4.475 eV) was conducted to determine Φ_gold tip_.

PEC performance characteristics of deposited thin films were tested in a home-built, single-absorber test cell. The cell body consisted of PEEK and a quartz window for sample illumination. The films grown on Si were configured as working electrodes (WEs), whereas a Pt wire and 3 M KCl Ag/AgCl served as counter (CE) and reference electrode (RE), respectively. The WEs were prepared in a lollipop configuration, where the Si backside was first scratched to remove the native oxide layer, and a Ga–In eutectic mixture (Alfa Aesar) was applied to ensure good electric contact to a Cu wire that was glued onto the backside of the Si wafer using a two-component silver epoxy (Circuit Works, CW2400). After inserting the Cu wire into a glass tube, the sample backside and glass tube were sealed with an insulating and chemically stable dark epoxy resin (silicon polymer compound, 101RF BLACK, Microset). The electrodes were immersed in an Ar-saturated 1 M NaOH electrolyte (pH 14) and connected to a Biologic potentiostat (SP-300). Sample illumination was realized with a research-grade LED AM 1.5G solar simulator (Pico, G2 V) at 100 mW/cm^2^. This illumination was manually chopped for linear sweep voltammetry (LSV) measurements, whereas it was kept constant during illuminated chronoamperometry (CA) measurements. Each sample was measured following a uniform experimental protocol: First, for conditioning CVs, the voltage was cycled four times between 0.25 and 1.0 V vs reversible hydrogen electrode (RHE) with a scan rate of 20 mV/s without illumination of the sample. A second set of CV scans, identical to the first, was conducted under illumination (AM 1.5G at 100 mW/cm^2^). Three consecutive, identical LSV measurements were then performed, starting at the open circuit potential, *E*_OC_, and sweeping to 1.6 V vs RHE at a scan rate of 20 mV/s, during which the current response was recorded while the illumination was chopped to test the photoresponse.

## Results and Discussion

Zr_*x*_O_*y*_N_*z*_ thin films were synthesized by reactive magnetron sputtering on fused silica and on (100) n^+^-Si wafers. The O and N contents of the films were carefully controlled by a stepwise increase of the reactive O_2_ gas flow (0, 0.08, 0.12, 0.16, 0.20, 0.24, 0.28, and 0.32 sccm) while maintaining constant Ar and N_2_ flows of 10 and 20 sccm, respectively. All films have similar thicknesses, between 60 and 80 nm, as determined by XRR measurements. Throughout this work, the films are denoted by the O_2_ flow rate used during deposition. Digital photographs of the sample series are provided in Figure S1.

**Figure 1:**
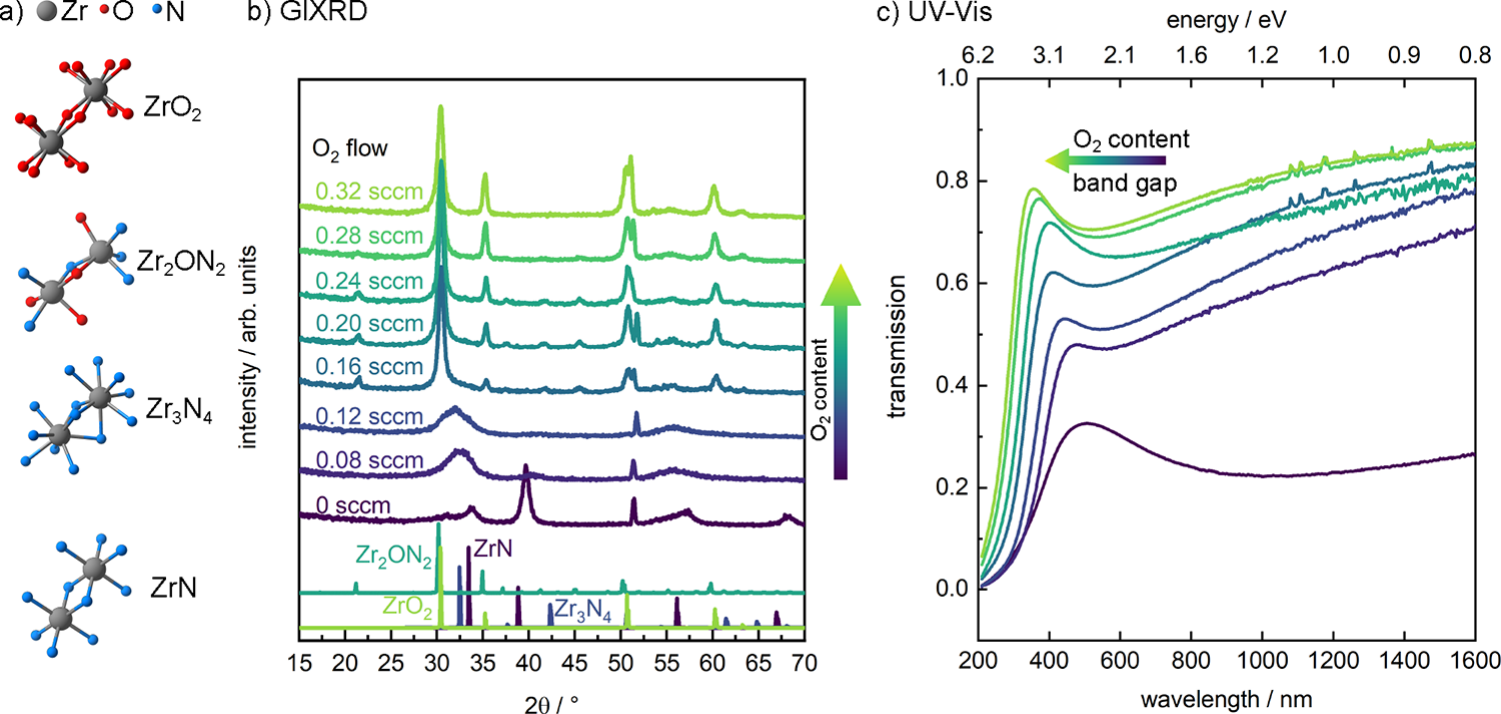
(a) Structural motifs of two neighboring Zr atoms within ZrN, cubic Zr_3_N_4_, bixbyite-type Zr_2_ON_2_, and fluorite-type ZrO_2_. Only portions of the unit cell are shown for better visualization of the Zr–O–N coordination. Zr atoms are shown in gray, oxygen in red, and nitrogen in blue, generated with VESTA.^26^ (b) Grazing incidence X-ray diffraction (GIXRD) patterns of sputter-deposited Zr_*x*_O_*y*_N_*z*_ films, along with reference patterns of ZrN (dark violet), Zr_3_N_4_ (dark blue), bixbyite-type Zr_2_ON_2_ (turquoise), and fluorite-type ZrO_2_ (green). Films were deposited with variable amounts of oxygen (as indicated in the legend on the left) and a constant flow of 20 sccm N_2_ and 10 sccm Ar. At approximately 52°, a reflection of the crystalline Si(100) substrate is visible; all other reflections can be attributed to the different Zr_*x*_O_*y*_N_*z*_ crystalline phases. (c) UV–vis transmission spectra corresponding to the films shown in panel b.

Figure 1a shows structural motifs of the Zr_*x*_O_*y*_N_*z*_ system, along with the experimentally determined evolution of the thin film structures (Figure 1b) and optical properties (Figure 1c) obtained by GIXRD and UV–vis measurements, respectively. For this sample library, with increasing oxygen content, we observe a transition from crystalline ZrN (0 sccm O_2_) to highly disordered Zr_3_N_4_ (0.08, 0.12 sccm O_2_) to crystalline bixbyite-type Zr_2_ON_2_ (0.16, 0.20, 0.24 sccm O_2_) to crystalline fluorite-type ZrO_2_ (0.28, 0.32 sccm O_2_), as shown in Figure 1b for films grown on Si substrates. An equivalent structural evolution is observed for the films grown simultaneously on fused silica (Figure S2). These observations align with previous studies that have used reactive sputtering with different oxygen-to-nitrogen ratios to prepare Zr_*x*_O_*y*_N_*z*_ thin films.^12,29−31^ However, although we succeeded in growing crystalline phases of ZrN, Zr_2_ON_2_, and ZrO_2_, we could not identify conditions under which crystalline Zr_3_N_4_ could form. This finding is consistent with the extreme conditions that are typically required to synthesize Zr_3_N_4_, such as high-pressure, high-temperature growth of powders^32^ and filtered cathodic arc deposition of films.^33^ To assess the as-grown film morphology, we used SEM (Figure S3). All deposited films are closed and continuous. With increasing oxygen content in the sputter gas mixture, the film morphology evolves from round to needle-like grains, where the average grain size continuously increases from approximately 12 to 22 nm.

Corresponding optical transmission measurements (Figure 1c) indicate the semiconducting or insulating nature of all but one metallic sample (ZrN, 0 sccm O_2_), with an absorption edge that shifts toward shorter wavelengths as the oxygen content in the gas mixture increases. Although the ZrN film obtained with 0 sccm O_2_ shows a metallic appearance, it exhibits greater than 20% optical transmission at longer wavelengths, which is likely due to the inclusion of oxygen impurities. Consistent with prior reports,^34^ we find that oxygen incorporation can be nearly completely suppressed via application of a substrate bias during deposition (not shown). However, because the present focus is on higher oxygen content materials, no substrate bias was applied for the films investigated here. Using Tauc analysis of the recorded transmission spectra, we estimated the band gaps of the Zr_*x*_O_*y*_N_*z*_ thin films. Although we acknowledge that Tauc analysis was originally developed for amorphous materials^35^ and has its limitations,^36^ its systematic application offers insights into how the optical band gap evolves with varying oxygen contents. The Tauc plots corresponding to the sample series shown in Figure 1c are given in Figure S4, and the determined band gaps are reported in Table S1. The analysis indicates that the optical band gap increases with increasing oxygen content. For all of the nonmetallic films, the observed band gaps are in the UV–visible range. We note that the obtained band gap values for films showing Zr_2_ON_2_ structure (samples 0.16–0.24 sccm O_2_) are larger than those reported in literature, which were derived from Zr_2_ON_2_ powders.^21^ This discrepancy likely stems from subtle variations in oxygen contents among the considered materials, thin film interference effects, and the limitations of the Tauc method^36^ that complicate the analysis in the present case.

We now consider the relationships between structural and optical properties of the films as a function of oxygen content in more detail. The controlled introduction of oxygen into the reactive sputter gas mixture leads to a modified coordination environment around zirconium, as indicated by the structural motifs associated with their respective crystal structures shown in Figure 1a. Introduction of small quantities of oxygen into the reactive gas mixture (0.08, 0.12 sccm O_2_) helps bring the metal cation into the fully oxidized Zr^4+^ state, resulting in a highly defective Zr_3_N_4_-like structure. Such an oxygen-inductive effect has also been observed for tantalum nitrides and can generally be used to promote the formation of nitrogen-rich metal nitride compounds.^37,38^ This nitrogen enrichment, along with the high oxidation state of Zr, leads to the opening of a band gap, as indicated in the transmission spectra shown in Figure 1c. As more oxygen is introduced into the reaction gas mixture, crystalline thin films of bixbyite-type Zr_2_ON_2_ and fluorite-type ZrO_2_ emerge. Because the bixbyite- and fluorite-type crystal structures are closely related, they show similar diffraction patterns. The primary difference between these structures is that for bixbyite-type Zr_2_ON_2_ (0.16, 0.20, 0.24 sccm O_2_), a quarter of the anion positions of the fluorite-type structure are unoccupied. Previous reports on Zr_2_ON_2_ using neutron diffraction have shown that oxygen and nitrogen randomly populate the occupied anion sites.^39^ The reduced symmetry associated with the structural vacancies on the anion sites leads to the appearance of weak reflections at 21.4, 37.5, 41.7, and 45.5°. The intensities of these reflections diminish at higher oxygen contents (0.28, 0.32 sccm O_2_) because of the filling of structural anion vacancies with oxygen, resulting in conversion from the bixbyite- to the fluorite-type ZrO_2_ structure, showing only the primary XRD reflections at 30.5, 35.3, 50.8, and 60.5°. Interestingly, the presence of nitrogen during the sputtering process appears to stabilize fluorite-type ZrO_2_. When sputtering under similar conditions in a pure Ar/O_2_ mixture, a mixed film of monoclinic baddeleyite-type and cubic fluorite-type ZrO_2_ evolves (Figure S5).

The variable band gap within the deposition series aligns with expectations for transitioning from a TM nitride via a TM oxynitride to a TM oxide. Because the valence band of these materials is dominated by N 2p and/or O 2p states, increasing oxygen incorporation leads to a shift of the valence band maximum toward lower energies. The conduction band minimum, however, is dominated by Zr 4d states and, hence, is less affected by the compositional change on the anion positions. Based on our recorded transmission spectra in Figure 1c, we indeed observe a trend of a gradually increasing band gap with increasing oxygen content in the reaction gas mixture, in line with previous literature.^31^

**Figure 2:**
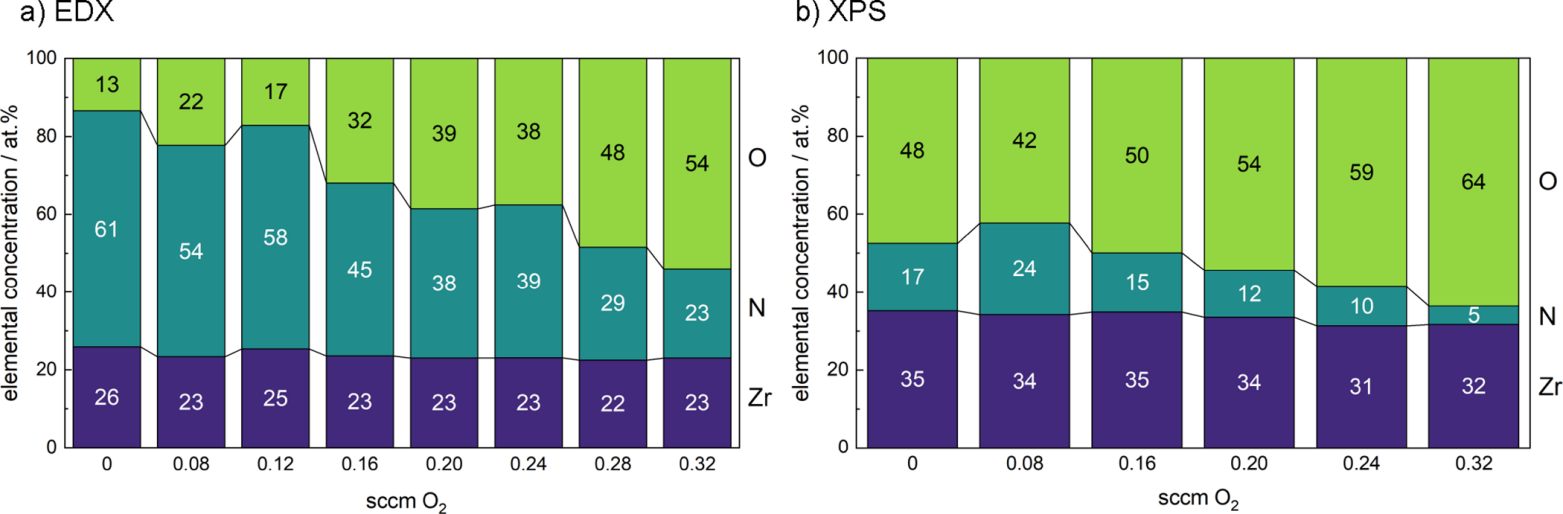
Elemental composition of the Zr_*x*_O_*y*_N_*z*_ films based on (a) energy-dispersive X-ray spectroscopy (EDX) and (b) X-ray photoelectron spectroscopy (XPS).

To gain further insight into the role of anion compositions on the properties of Zr_*x*_O_*y*_N_*z*_ films, we assessed their bulk and surface elemental content using EDX and XPS, respectively. As shown in Figure 2, both techniques reveal a nearly constant Zr content across the complete sample series. In addition, we observe the expected trend of increasing O and decreasing N contents in the films with increasing O_2_ concentration in the sputter gas mixture. The absolute values of the Zr contents obtained by EDX and XPS differ by roughly 10%, which is due to a systematic overestimation of light elements, such as O and N, with EDX.^40^ However, as will be shown later for similar films, the Zr concentration obtained from XPS agrees well with the Zr content obtained from ERDA measurements. In both EDX and XPS, relative changes in concentration can be determined much more precisely than absolute values. Nevertheless, the oxygen contents obtained with XPS are generally significantly higher than those determined by EDX, especially for nitrogen-rich samples, suggesting that Zr nitride and oxynitride films are prone to surface oxidation upon exposure to ambient conditions.^41^

**Figure 3:**
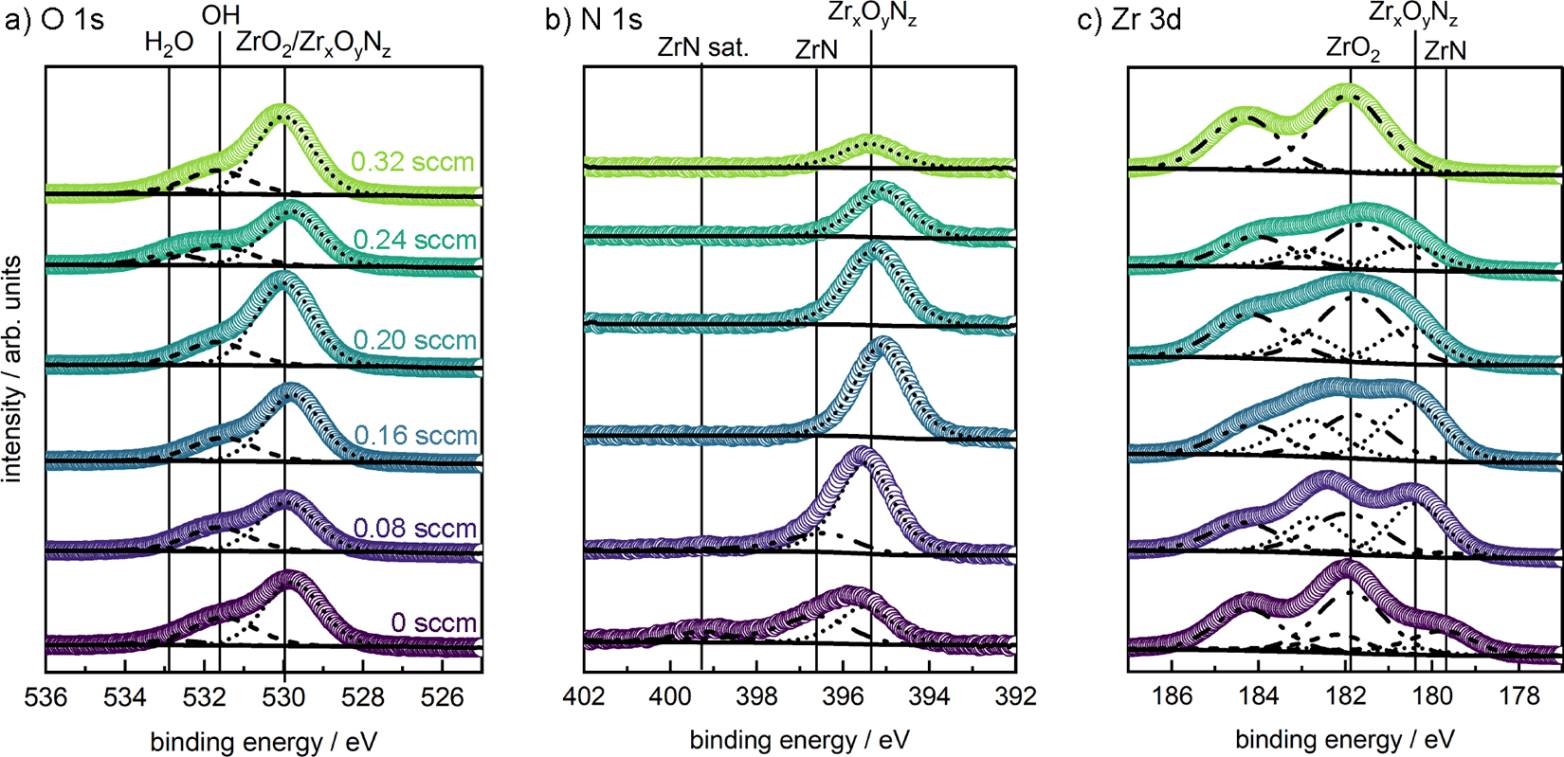
XPS spectra of the (a) O 1s, (b) N 1s, and (c) Zr 3d core-level regions of a selection of the deposited Zr_*x*_O_*y*_N_*z*_ films (the oxygen content in the reactive sputter gas mixture is indicated in panel a). Identified species are marked with vertical lines. In the O 1s spectrum, it is not possible to deconvolute the contribution of ZrO_2_ and Zr_*x*_O_*y*_N_*z*_.

Additional information regarding the film composition, including surface oxidation, is provided by analysis of individual XPS core levels. As shown in Figure 3a, the O 1s spectral shapes are largely unaffected by the oxygen content within the reactive sputter gas. In particular, all spectra show a primary contribution at 530.0 eV. Although this component can be assigned to O within Zr_*x*_O_*y*_N_*z*_ and ZrO_2_, individual contributions from each of these two phases cannot be resolved from one another with our experimental setup. In addition, we observe a contribution from surface OH groups, potentially convoluted with carbon–oxygen bonds of adsorbed carbon species at 531.7 eV and a small contribution of adsorbed H_2_O at 532.9 eV. The N 1s spectra, shown in Figure 3b, initially show a contribution of ZrN (0 and 0.08 sccm O_2_) that disappears for higher oxygen flows. All samples show Zr_*x*_O_*y*_N_*z*_, which first increases from 0 to 0.16 sccm O_2_ and then decreases at higher oxygen flows. From these XPS spectra, we cannot distinguish between nitrogen in the disordered Zr_3_N_4_ and the crystalline Zr_2_ON_2_; hence, they are jointly labeled as Zr_*x*_O_*y*_N_*z*_.

A comparison of the Zr 3d spectra (Figure 3c) indicates trends that are similar to those observed by GIXRD, with increasing oxygen content in the reaction gas mixture leading to a gradual transition from ZrN (197.7 eV) via Zr_*x*_O_*y*_N_*z*_ (180.3 eV) to ZrO_2_ (181.9 eV). Although the included spectral deconvolution serves as an informative indicator for the evolution of composition and binding, the exact deconvolution is nonambiguous based on the broad line width of our nonmonochromatic X-ray source and the spectral overlap between the individual components. Interestingly, this deconvolution indicates that the 0 sccm O_2_ sample, which comprises ZrN, shows a more significant contribution from ZrO_2_ than the 0.08 and 0.16 sccm O_2_ samples, which are composed of Zr_3_N_4_ and Zr_2_ON_2_, respectively. From this observation, we can deduce that metallic ZrN is even more prone to surface oxidation than the semiconducting Zr_*x*_O_*y*_N_*z*_ films.

In general, we observe subtle, nonmonotonic binding energy shifts in the components of the individual core levels (O 1s vs N 1s vs Zr 3d). The reason for these shifts is not yet fully understood and is the subject of a current study within our group on a related materials system. However, we note that subtle changes in the Fermi level position, along with variable anion–cation orbital overlaps, may lead to a complex behavior as a function of oxynitride composition.

**Figure 4:**
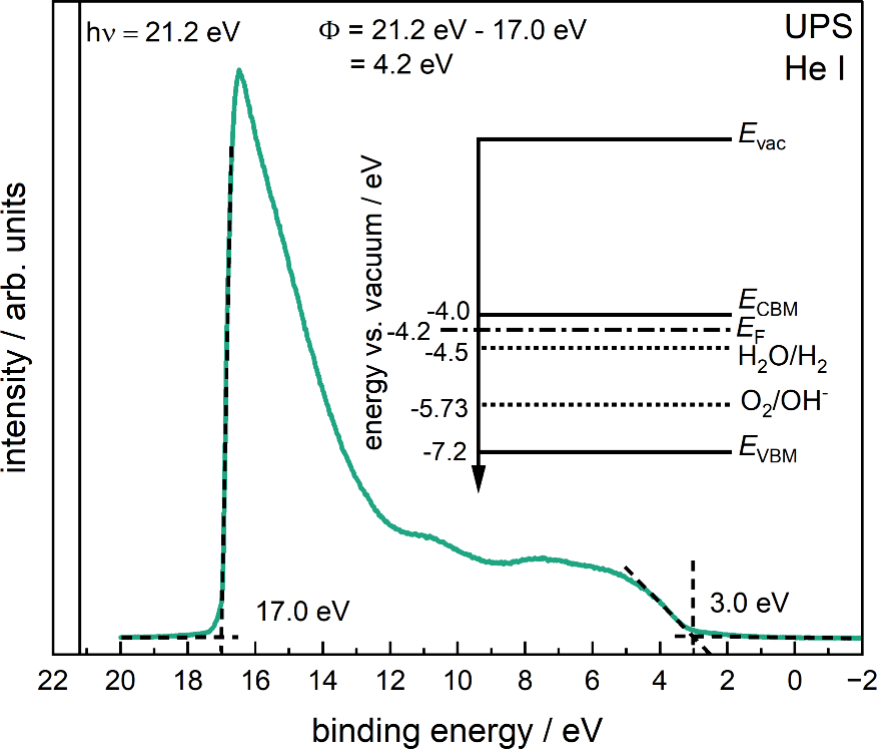
Ultraviolet photoelectron spectroscopy (UPS) measurement of a representative Zr_2_ON_2_ thin film using the He I emission line. The secondary electron cutoff (SECO) and the valence band maximum (VBM) positions are indicated by dotted, intercepting lines, along with the respective values. The work function, Φ, is calculated by the difference of the He I emission line energy and the SECO. The inset shows the energy level diagram of the system, where *E*_vac_ marks the vacuum level, *E*_CBM_ is the conduction band minimum, *E*_F_ the Fermi level, *E*_VBM_ is the valence band maximum, and, for comparison, H_2_O/H_2_ and O_2_/OH^–^ represent the water reduction and oxidation potentials, respectively.

To assess the energy band alignment of our Zr_2_ON_2_ thin films relative to the water reduction and oxidation potentials, we performed UPS measurements (Figure 4). Based on the small kinetic energy of the emitted photoelectrons, UPS is highly surface sensitive and only probes the top atomic layers. Hence, for the case of our Zr_2_ON_2_ thin films, UPS mainly probes the properties of the oxygen-enriched surface layer that, during water splitting, is in direct contact with the electrolyte. Furthermore, we note that because of surface band bending, the band alignment of the bulk material might be slightly different than at the surface. Nevertheless, based on the work function and the valence band maximum determined from the UPS measurements, along with the approximate band gap obtained from Tauc analysis, we constructed an energy level diagram of Zr_2_ON_2_ with respect to the vacuum level, as well as the water oxidation and reduction potentials (inset of Figure 4). From this diagram, the generally favorable energy level alignment of Zr_2_ON_2_ with respect to the water redox potentials becomes evident: whereas the Fermi level and conduction band minimum of the n-type semiconductor Zr_2_ON_2_ are at more negative potentials (higher in energy) than the water reduction potential, the valence band maximum is at a more positive potential (lower in energy) than the water oxidation potential. Hence, the energy levels of Zr_2_ON_2_ straddle the water redox potentials, providing a driving force for the charge transfer of photoinduced charge carriers.

**Figure 5:**
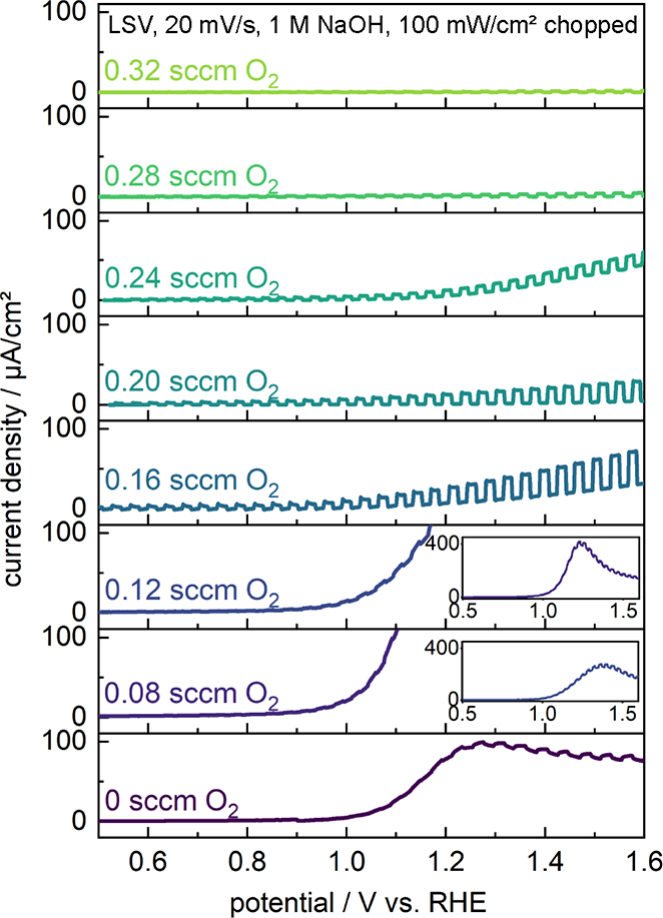
Linear sweep voltammograms (LSVs) of different Zr_*x*_O_*y*_N_*z*_ films on Si substrates deposited with varying oxygen contents in the reactive gas mixture as indicated in the figure (as in Figure 1). The LSVs were recorded in 1 M NaOH under chopped front-side illumination (AM 1.5G, 100 mW/cm^2^). The insets for the 0.08 and 0.12 sccm O_2_ samples show the zoomed-out versions of the LSVs to demonstrate the film self-oxidation peaks at 1.3 and 1.2 V vs RHE, respectively.

Having established the structural, compositional, electronic, and optical properties of the series of Zr_*x*_O_*y*_N_*z*_ samples, we now turn to an assessment of their PEC activities. Linear sweep voltammetry (LSV) measurements were conducted using a three-electrode setup in 1 M NaOH (pH 14) under chopped illumination without adding a hole scavenger. As shown in Figure 5, significant differences in the PEC characteristics are observed as a function of oxygen content within the films. For the metallic ZrN and disordered Zr_3_N_4_ samples grown with the lowest O_2_ flows (0, 0.08, and 0.12 sccm), a steep increase of the dark current is observed at potentials above approximately 1.0 V vs RHE. Although these films are slightly photoactive at more anodic potentials, the overall current decreases with increasing potential, indicating that the samples undergo severe surface oxidation. This conclusion is supported by the subsequent collection of additional LSVs that show significantly lower overall currents, with only slight photoactivity (not shown). As described above, XPS analysis indicated that these nitrogen-rich samples are especially prone to surface oxidation. Thus, we conclude that formation of a comparatively wide band gap zirconium oxide phase on the surface presents a barrier to interfacial charge injection and, thus, greatly reduced current densities. Furthermore, PEC characterization of the nitrogen-doped ZrO_2_ samples produced with the highest O_2_ flows (0.28 and 0.32 sccm O_2_) reveal a negligible photoresponse, suggesting that surface oxide layers formed in situ are also unlikely to be photoactive under simulated solar illumination, likely due to their wide band gaps.

In contrast to the pure nitride and oxide phases presented above, the crystalline bixbyite-type Zr_2_ON_2_ films (0.16, 0.20, 0.24 sccm O_2_) exhibit a measurable photoresponse, with photocurrents on the order of 10 μA/cm^2^ at 1.23 V vs RHE, along with concomitant dark currents on the order of tens of μA/cm^2^. The observed dark current likely stems from the oxidation of zirconium nitride defect phases in the near-surface region. The dark current decreases in consecutive LSV scans (not shown), indicating that these regions become passivated with zirconium oxide layers under anodic conditions. We note here that we also performed chopped LSV measurements after adding 0.1 M H_2_O_2_ as a hole scavenger to the electrolyte (see Figure S6). Remarkably, the addition of the hole scavenger did not increase the measured photocurrent, alluding to the native catalytic activity of the Zr_2_ON_2_ thin films for water oxidation. Within the sample series, the photoactivity is highest for the 0.16 sccm O_2_ sample and gradually decreases as more oxygen is added to the deposition gas mixture. In contrast to the pure nitride films, LSVs of these oxynitride samples do not show strong indications for surface oxidation. In addition, we note that increasing oxygen content in the reactive sputter gas could also lead to deleterious oxidation of the Si substrate, thereby increasing the back-contact resistance. Although electrical contact optimization is beyond the scope of the present work, integration of electron-selective contacts or interfacial TCOs for improved majority carrier extraction may provide a viable route to improving PEC performance characteristics. A recent review on the substrate choice for photoanodes can be found in Hou et al.^42^

To further investigate the stabilities of all samples, CA measurements were performed under constant illumination and an applied bias of 1.23 V vs RHE (Figure S7). Although these measurements confirm that the oxynitride films are more stable than the pure nitride films, all samples undergo degradation under sustained operation. In particular, whereas the pure nitride films degrade within a few seconds, the current densities of the oxynitride films more slowly decrease over the course of the 15 min CA test, reducing to less than 0.1 μA/cm^2^ after 15 min, even for the most active sample. Overall, we see a more pronounced degradation for the sample possessing the highest initial photoactivity (0.16 sccm O_2_) compared to the more oxygen-rich oxynitride films. This observation suggests that the stability of the Zr–O–N system can be increased with higher oxygen content, although this comes at the cost of reduced photoactivity. Nevertheless, corrosion protection strategies or the integration of cocatalysts would be necessary to ensure long-term durability.

**Figure 6:**
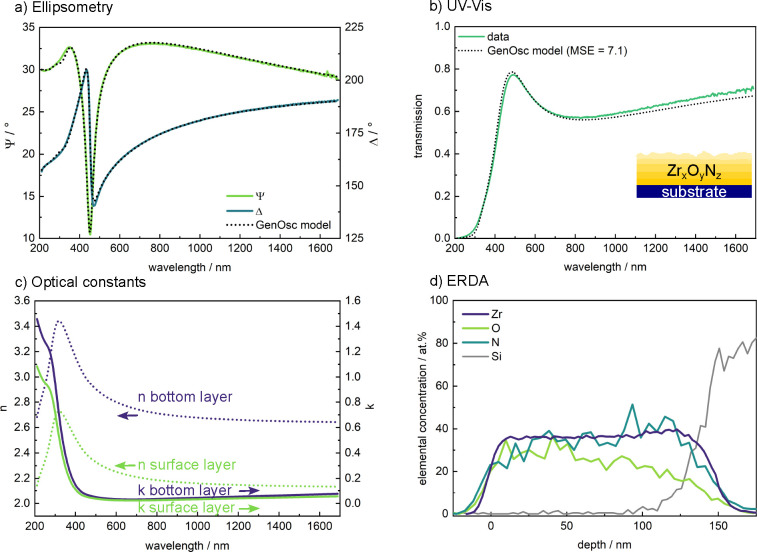
Optical and compositional analysis of a representative Zr_2_ON_2_ thin film. (a) Variable angle spectroscopic ellipsometry data (for clarity, presented for a single angle of 45°), along with the fit obtained using a general oscillator (GenOsc) model. (b) Corresponding transmission data and predicted transmission spectrum based on the GenOsc model derived from the ellipsometry data in panel a. The inset in panel b shows a model of the sample. The best fit was obtained for a graded layer of Zr_*x*_O_*y*_N_*z*_ on a SiO_2_ substrate. (c) Representative refractive index, *n*, and extinction coefficient, *k*, obtained for the top and bottom layers of the GenOsc model in panel a. Reduced *n* and *k* values are indicative of a higher oxygen content. (d) Elastic recoil detection analysis (ERDA) measurements of the same sample. Note that the depth scale in nm is only an approximation based on the calculated film density.

Overall, the PEC characteristics confirm that Zr oxynitrides are photoactive, but the photocurrent densities are extremely low compared to state-of-the-art thin film photoanodes.^43^ To better understand the origin of this poor PEC performance, we further investigated the optical properties of the semiconducting bixbyite-type Zr_2_ON_2_ phase via variable angle spectroscopic ellipsometry (VASE). Figure 6a shows representative VASE data from a Zr_2_ON_2_ film grown with 0.24 sccm O_2_ flow, with one of the several measurement angles (45°) presented for clarity. We note that, compared to the first sample library reported above, this film was grown with a reduced working distance between the sputter target and substrate but was confirmed to yield similar crystallinity.

The complete set of experimental ellipsometry data was analyzed by applying a Kramers–Kronig consistent GenOsc model, as described in the Experimental Section. Importantly, we obtained the best fits using a graded Zr_*x*_O_*y*_N_*z*_ layer, which reduces the root-mean-square error by almost an order of magnitude compared to a uniform film model. From this VASE model, the corresponding UV–vis transmission was computed and is compared to the experimental measurement in Figure 6b. The prediction and measurement closely agree, confirming that the modeled graded layer configuration (schematically shown in the inset in Figure 6b) describes the film properties well. Based on this graded model, the optical constants of the film are predicted to vary throughout the film thickness. Figure 6c shows the obtained refractive index, *n*, and extinction coefficient, *k*, for the region at the interface to the substrate and at the top surface of the film. These functions thus represent the range of variation of the optical constants across the film thickness. Overall, we find that the optical constants are larger near the bottom of the film and gradually decrease toward the top surface and that they are slightly higher compared to previous reports on reactively sputtered Zr_*x*_O_*y*_N_*z*_ thin films.^44^

Considering that the optical constants of ZrO_2_ are known to be smaller than those of Zr_*x*_O_*y*_N_*z*_,^45^ a possible origin of the graded optical properties could be surface oxygen enrichment in the films. To test this hypothesis, ERDA measurements were performed on the same sample. As shown in Figure 6d, this elemental analysis confirms the presence of a gradient in the film composition that is consistent with the observed gradient of optical characteristics. Whereas the Zr content remains approximately constant throughout the sample thickness, we observe an increase in the O and a decrease in the N content from the bottom interface toward the surface of the film. In terms of absolute composition, the ERDA measurements agree well with the Zr content of roughly 34 at. % determined by XPS, confirming that the lower Zr content obtained from EDX stems from an underestimation of Zr relative to O and N contents.

Importantly, the observed O and N gradients within our films may create an unfavorable energetic landscape for charge carrier separation and hole extraction in photoanodes. In particular, the increasing oxygen content toward the upper surface of the film is expected to shift the valence band maximum downward, introducing an energetic gradient that opposes hole extraction. The specific origin of the observed gradient is not yet clear. However, one hypothesis is that, despite waiting for steady-state conditions to be reached in the sputtering chamber prior to opening the substrate shutter, metallic Zr that remains on the chamber walls from the target conditioning period might initially act as oxygen getter material, leading to a reduced oxygen concentration in the initial reaction gas mixture. Another possibility is that when the films are exposed to air, oxygen from the atmosphere not only oxidizes the surface but also diffuses into the film, leading to the observed O gradient. The former hypothesis appears more likely given the reported self-passivating nature of ZrO_2_ surface oxide layers under atmospheric conditions.^46^

Considering that increasing oxygen content toward the surface of the films is likely to create an internal electric field distribution that opposes hole separation and extraction at the solid|liquid interface of oxynitride photoanodes, we attempted to apply postdeposition annealing treatments that could eliminate the compositional gradient. Indeed, postdeposition annealing is a commonly used strategy to improve the PEC performance of semiconductor photoelectrodes.^37,47,48^ For this purpose, we first investigated several thermal treatments using different annealing systems, including both rapid thermal (RTA) and conventional annealing in H_2_, as well as ammonolysis under NH_3_ flow in a tube furnace. However, we found that all films were converted into transparent zirconium oxides upon annealing at temperatures higher than 450 °C despite the reducing annealing atmospheres. We note that similar thermal treatments have been applied to tantalum nitrides in the same annealing ovens without such oxidation occurring,^37,47^ thus highlighting the challenge of working with highly oxophilic Zr oxynitrides. Indeed, even small concentrations of residual oxygen or water in otherwise reducing atmospheres are sufficient to oxidize the zirconium oxynitride thin films when heated above 450 °C.

To circumvent the observed oxidation of films during reactive annealing, we next attempted an annealing procedure under high-vacuum conditions (10^–8^ mbar) in the sputter system itself. In particular, after initial deposition and exposure to air, the samples were reintroduced into the sputter system and annealed at 700 °C for 40 min or at 750 °C for 80 min. AFM and SEM measurements reveal that the grain sizes and morphologies are not significantly affected by annealing (Figures S8 and S9). CPD measurements in air show that the work function also remains unchanged, indicating an unaltered position of the Fermi level. Furthermore, GIXRD and UV–vis measurements of these samples revealed that they retained their initial bixbyite-type structures and band gaps (Figure S10), respectively, and did not undergo bulk oxidation, thus motivating further investigations of their physical and functional properties.

Comparative ERDA measurements of the as-grown film and the sample annealed at 750 °C for 80 min (Figure S11) indicate minimal redistribution of oxygen and nitrogen within the films. In particular, both samples are characterized by the previously described anion concentration gradients, with the oxygen (nitrogen) content increasing (decreasing) toward the surface. Although the bulk composition profile is unaffected by the annealing procedure, ERDA measurements show a steep increase in the near-surface oxygen content and a concomitant decrease in the nitrogen concentration that is not as pronounced for the as-grown sample. As shown in Figure S12, XPS measurements confirm this oxygen enrichment at the surface following high-vacuum annealing, which is especially apparent from analysis of the Zr 3d XPS core-level spectra (Figure S12c). Compared to the as-grown sample, which has approximately equivalent spectral contributions from Zr_*x*_O_*y*_N_*z*_ and ZrO_2_, the ZrO_2_ contribution becomes dominant for the sample annealed at 750 °C for 80 min. Reducing the annealing temperature and time to 700 °C for 40 min leads to less significant surface oxidation and an intermediate surface composition. In summary, we conclude that high-vacuum annealing up to 750 °C is insufficient to eliminate the subsurface anion composition gradient and can lead to surface oxidation, which is more pronounced at higher annealing time and temperature.

**Figure 7:**
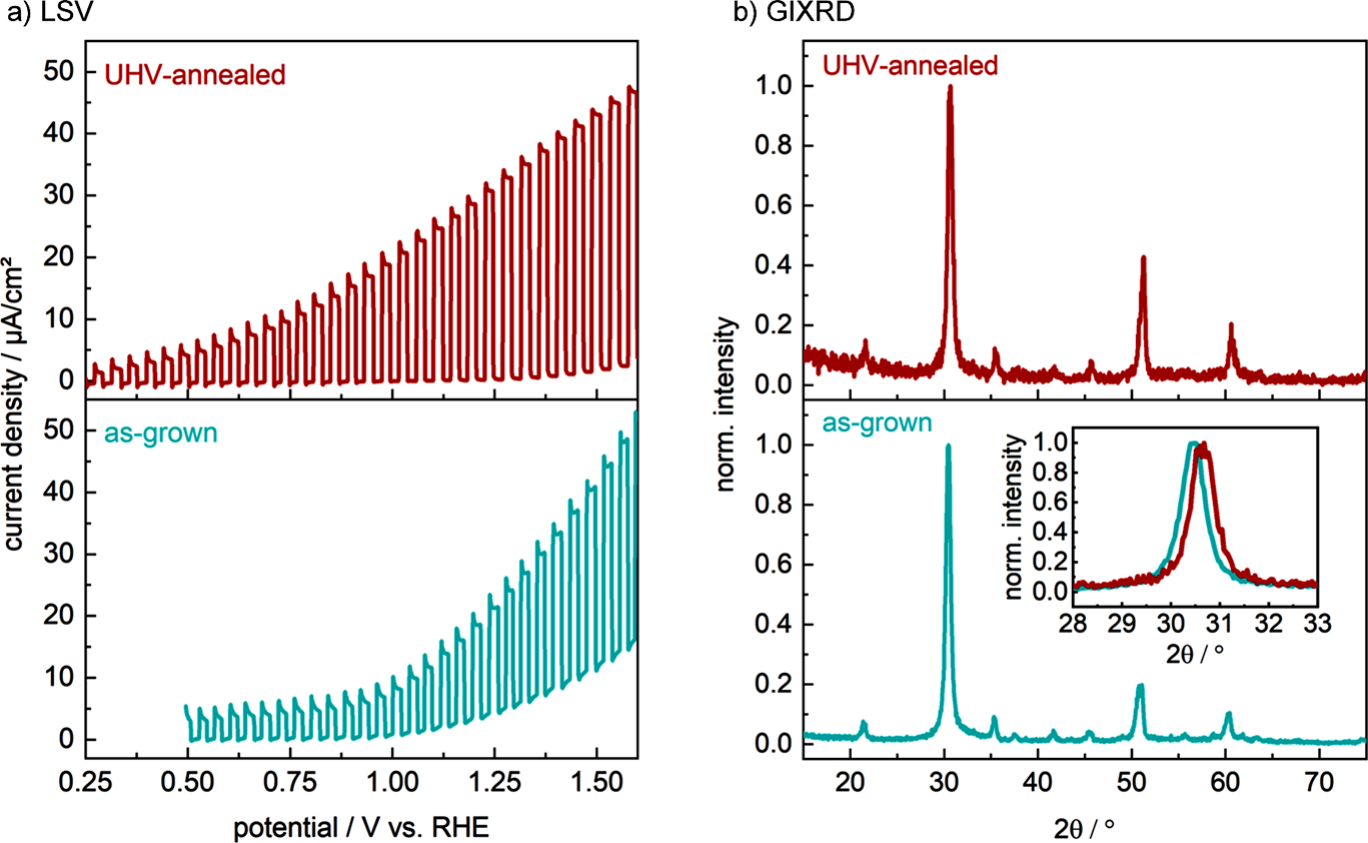
(a) Linear sweep voltammograms (LSVs) and (b) grazing incidence X-ray diffraction (GIXRD) patterns of Zr_*x*_O_*y*_N_*z*_ films on Si substrates, (bottom) as-deposited and (top) following postsynthetic high-vacuum annealing at 700 °C for 40 min. The LSVs were recorded in 1 M NaOH under chopped front-side illumination (AM 1.5G, 100 mW/cm^2^) at a sweep rate of 20 mV/s. High-vacuum annealing results in increased photocurrent density, decreased dark current density, and a beneficial cathodic shift of the open circuit potential (from which the LSVs were initiated). The inset in panel b shows a zoomed-in comparison of the most intense reflection of the as-grown and UHV-annealed samples at 30.5°. Annealing does not significantly affect the fwhm of the diffraction patterns but leads to a shift of the reflection angle by 0.2° toward higher angles.

Although high-vacuum annealing did not eliminate the undesired composition gradients within the films, we proceeded to compare the PEC performance characteristics before and after thermal treatment. Here, the intermediate treatment of 700 °C for 40 min was selected to balance possible positive impacts of thermal annealing with detrimental surface oxidation that dominates at higher temperatures. Remarkably, as shown in Figure 7a, vacuum annealing of a Zr_2_ON_2_ film leads to enhanced PEC performance characteristics compared to the as-grown sample, with a lower onset potential (with 10 μA/cm^2^ reached at 0.75 V vs RHE compared to 1 V vs RHE, respectively), reduced dark current density (0.1 compared to 5 μA/cm^2^, respectively), and higher photocurrent density (30 compared to 15 μA/cm^2^, respectively) at 1.23 V vs RHE. Based on AFM, UV–vis, and CPD measurements, we can conclude that this enhanced performance is not a consequence of grain growth, band gap changes, or a modified energy level alignment. However, detailed comparison of the GIXRD patterns from the as-grown and annealed photoelectrodes (Figure 7b) reveals that the peak widths remain similar but the reflections shift toward higher angles, as highlighted in the inset of Figure 7b for the primary (222) Zr_2_ON_2_ reflection at ∼30.5°. Note that the slightly worse signal-to-noise ratio on the UHV-annealed XRD pattern stems from a smaller sample size. A similar shift upon thermal annealing has been previously observed for bixbyite-type Ta_2_N_3_ and was attributed to out-diffusion of interstitial oxygen impurities at structural vacancy sites of the bixbyite lattice.^37^ However, in the present work, no significant changes of the anion content were detected by ERDA. Furthermore, such a mechanism would be expected to yield increased diffraction intensity for the bixbyite-related (structural vacancy activated) features, which is not observed. Although these results may suggest that annealing results in strain relaxation or a local redistribution of anions, the exact mechanism of lattice contraction and its implication on charge transport are not yet known.

The pronounced reduction of the dark current following high-vacuum annealing suggests that conductive pathways to the surface are eliminated, thereby also positively enhancing photocurrent generation. Considering that the morphology, crystalline structure, and optical properties of the films are largely unaffected by annealing, these improvements to the functional PEC properties are likely a consequence of the observed surface oxidation. In this regard, we note that metallic nitride phases were found to be most susceptible to oxidation (see above), suggesting that any conductive minority phases near the surface or at grain boundaries would be preferentially oxidized, while still enabling interfacial hole transfer from the oxynitride photoanode to drive water oxidation. Although moderate surface oxidation can thus play a beneficial role in improving performance, severe surface oxidation to form a dense ZrO_2_ film would suppress charge transfer and PEC activity. In the present case, the observed increase in the photoresponse upon annealing indicates that mild surface oxygen enrichment outweighs this drawback. In general, these findings open up the possibility for engineering surface oxide layers on oxynitrides to beneficially suppress dark current and enhance photocurrent. In addition, this observation alludes to an appealing feature of oxynitrides as photoanode candidates: the formation of a self-passivating surface oxide layer. This self-passivation makes oxynitrides viable candidates to be combined with ultrathin atomic layer deposition protection layers, whose major drawbacks are pinholes that lead to corrosion of the underlying photoabsorbers. Although such pinholes can be detrimental to state-of-the-art III–V semiconductors,^49^ they can potentially be tolerated by oxynitride photoanode thin films that form stable surface oxide layers. However, for the thin films investigated here, elimination—or even reversal—of subsurface anion concentration gradients and band gap engineering will be essential for improving PEC activity.

## Conclusions

In this work, we have used reactive sputter deposition to grow a series of zirconium oxynitride thin films and investigated their suitability as semiconductor photoanodes for solar-driven water splitting. By introducing controlled amounts of oxygen at otherwise fixed deposition conditions, we observe a transition from metallic ZrN to disordered nitrogen-rich Zr_*x*_N_*y*_ to crystalline bixbyite-type Zr_2_ON_2_ to nitrogen-doped cubic ZrO_2_. Although both ZrN and the disordered nitrogen-rich Zr_*x*_N_*y*_ immediately oxidize when applying anodic potentials, introduction of additional oxygen into the films leads to a more stable crystalline structure (Zr_2_ON_2_), the opening of a band gap in the UV–visible range, and the emergence of photoelectrochemical activity. Based on chopped linear sweep voltammetry measurements, we show that Zr_2_ON_2_ films are photoactive in alkaline electrolyte with reasonably low onset potentials, indicating an overall favorable band alignment of the material with respect to the water oxidation and reduction potentials, which we confirm using ultraviolet photoelectron spectroscopy. Although the obtained photocurrent is low, the photoelectrochemical performance of the Zr_2_ON_2_ films can be enhanced via high-vacuum annealing, during which partial oxidation of the surface occurs, resulting in reduced dark current and improved charge transfer at the solid|liquid interface. However, the Zr_2_ON_2_ films are characterized by an internal composition gradient, with O increasing and N decreasing toward the surface. Considering that such composition changes lead to increased band gaps and energetically decreased valence band positions, we conclude that these gradients can impede hole separation and extraction, thus leading to low photocurrent responses. Such gradients may generally arise in reactively sputtered oxynitrides as chamber conditions evolve, and future efforts to actively prevent or even reverse these gradients could lead to improved photoanodic activity. Overall, we have grown dense and planar thin films that offer opportunities for band gap engineering through composition control and performance improvements through defect management. Hence, although the observed photocurrents are still considerably lower than for the benchmark photoelectrodes, further material and back contact optimization could potentially close this gap and provide Zr_2_ON_2_ as a functional and sustainable photoanode material.

## Supporting Information Available

Supporting figures of grazing incidence X-ray diffraction (GIXRD) on fused silica and for ZrO_2_ without nitrogen in sputter gas mixture, chronoamperometry under illumination, comparison of chopped linear sweep voltammetry with and without a hole scavenger, Tauc analysis of UV–vis data, digital photographs of samples, atomic force microscopy, scanning electron microscopy, GIXRD, UV–vis, elastic recoil detection analysis, and X-ray photoelectron spectroscopy measurements of samples subjected to different annealing treatments (PDF)
