# Supplementary material for: Zirconium Oxynitride Thin Films for Photoelectrochemical Water Splitting
Source: ACS Appl Energy Mater. 2024 Apr 27;7(9):4004–15. doi: 10.1021/acsaem.4c00303 (PMC11094725; doi:10.1021/acsaem.4c00303)
Supplement: Supplementary file 1 — ae4c00303_si_001.pdf [file ae4c00303_si_001.pdf]

Supporting Information

Zirconium Oxynitride Thin Films for Photoelectrochemical Water Splitting

Verena Streibel,[1,2,*] Johanna L. Schönecker,[1,2] Laura I. Wagner,[1,2] Elise Sirotti,[1,2] Frans Munnik[3], Matthias Kuhl,[1,2] Chang-Ming Jiang,[1,2] Johanna Eichhorn,[1,2] Saswati Santra,[1,2] Ian D. Sharp[1,2,*]

[1] Walter Schottky Institute, Technical University of Munich, 85748 Garching, Germany
[2] Physics Department, TUM School of Natural Sciences, Technical University of Munich, 85748 Garching, Germany
[3] Institute of Ion Beam Physics and Materials Research, Helmholtz-Zentrum Dresden-Rossendorf (HZDR), 01328 Dresden, Germany

*Corresponding authors e-mail: verena.streibel@wsi.tum.de, sharp@wsi.tum.de

Supplemental Figures

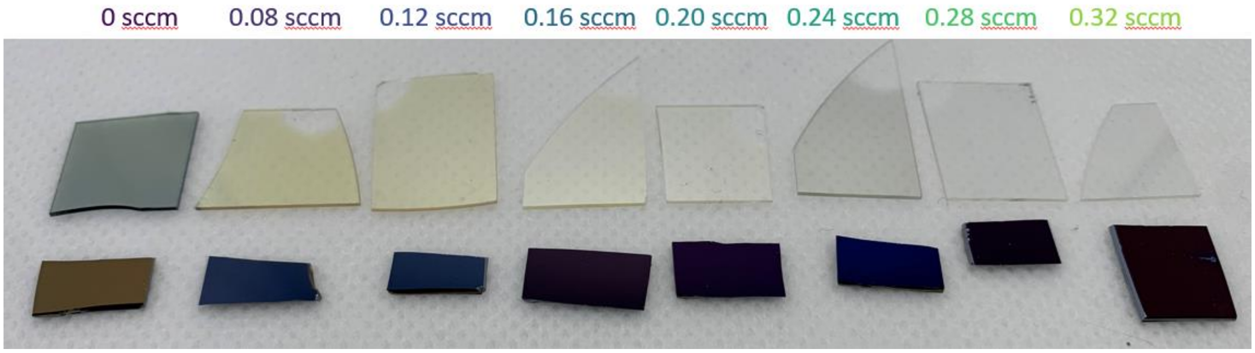

Figure S1. Photograph of the $Zr_xO_yN_z$ thin films on fused silica (top row) and n^+-Si (100) (bottom row). The oxygen flow used during deposition is given above the respective samples. The corresponding GIXRD and UV-vis data are provided in Figure 1b,c in the main text, and Figure S2.

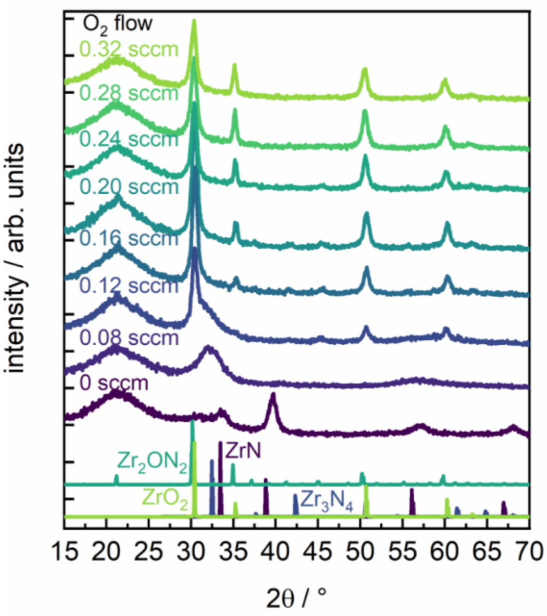

Figure S2. Grazing incidence X-ray diffraction (GIXRD) patterns of sputter-deposited $Zr_xO_yN_z$ films on fused silica substrates (simultaneously grown with those shown in Figure 1 in the main manuscript), along with reference patterns of ZrN (dark violet), Zr_3N_4 (dark blue), bixbyite-type Zr_2ON_2 (turquoise), and fluorite-type ZrO_2 (green). Films were deposited with variable amounts of oxygen (as indicated in the legend on the left) and a constant flow of 20 sccm N_2 and 10 sccm Ar.

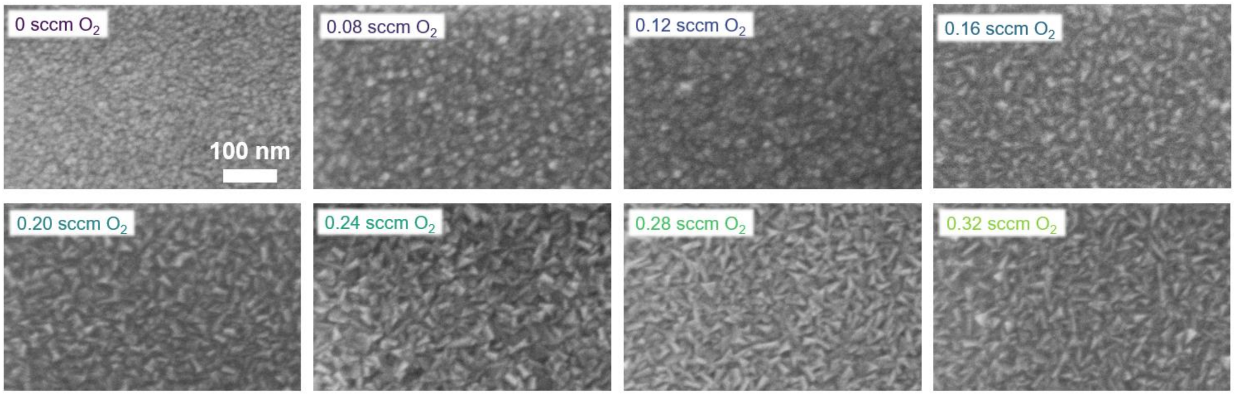

Figure S3. Scanning electron microscopy images of the as-grown samples deposited with 0 – 0.32 sccm O_2. The scale bar applies to all images. All films were deposited on n^+-Si(100) at 600 °C with variable amounts of oxygen and constant flows of 20 sccm N_2 and 10 sccm Ar at a Zr sputter power of 60 W.

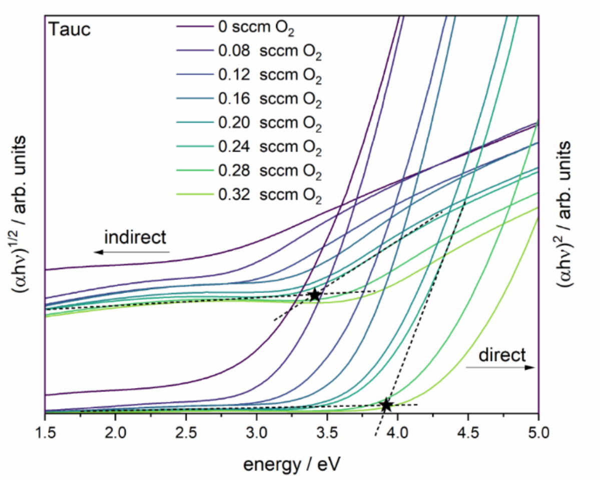

Figure S4. Tauc analysis was performed on the UV-vis data shown in Figure 1c in the main text. The determination of the approximate indirect and direct band gaps is exemplified on sample 0.24 sccm O_2 by dotted black lines, intersection points (representing the band gap energies) are highlighted by black stars. Given the limitations of Tauc analysis, thin film interference effects, and considerable sub-band gap absorption, the values are only approximate.

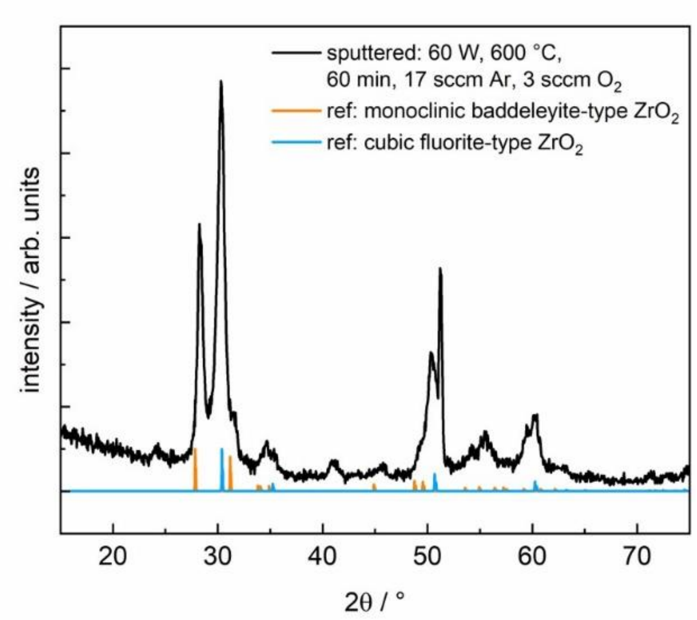

Figure S5. Grazing incidence X-ray diffraction (GIXRD) pattern of a sputter-deposited ZrO_2 film without the addition of nitrogen in the sputter gas mixture. The film consists of a mixture of baddeleyite- and fluorite-type ZrO_2.

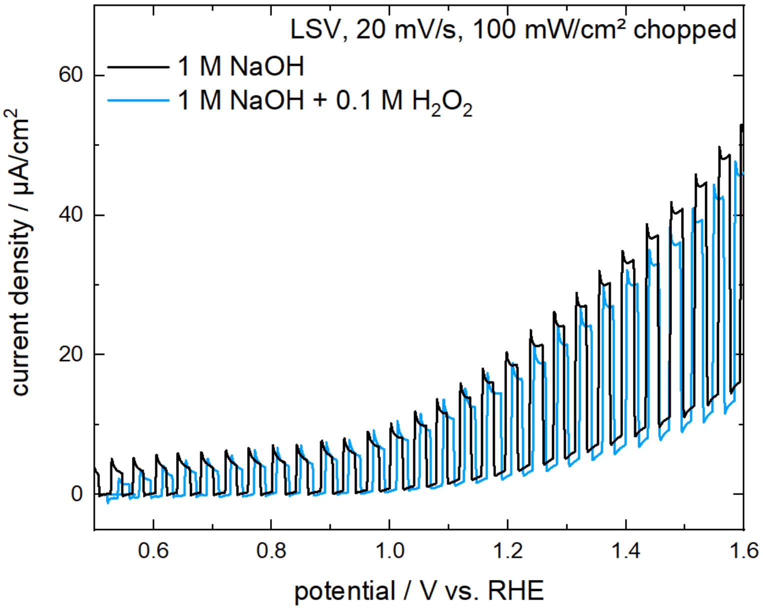

Figure S6. Linear sweep voltammograms (LSVs) of two identical Zr_2ON_2 films on Si substrates in 1 M NaOH and in 1 M NaOH + 0.1 M H_2O_2 acting as hole scavenger under chopped front-side illumination (AM 1.5G, 100 mW/cm²).

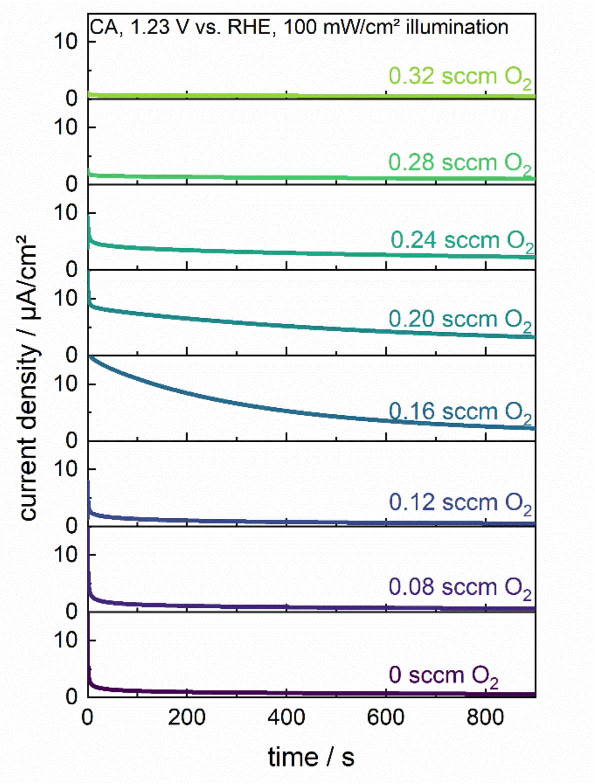

Figure S7. Chronoamperometry (CA) scans of different $Zr_xN_yO_z$ films on Si substrates, deposited with different oxygen contents as indicated in the label (reactive sputter mixture: 10 sccm Ar, 20 sccm N_2, x O_2). The CAs were recorded under constant front-side illumination (AM 1.5G, 100 mW/cm²) after a set of three chopped LSV scans. The CAs indicate that the films are not stable under photoelectrochemical conditions. Note that neither a catalyst nor a hole scavenger was added, so the observed degradation is expected.

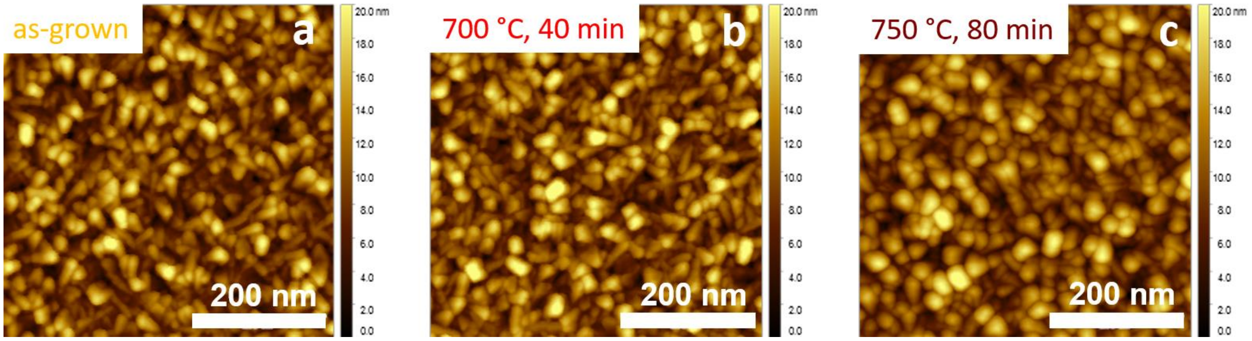

Figure S8. Atomic force microscopy (AFM) images of (a) an as-grown Zr_2ON_2, as well as films after post-synthetic annealing under high-vacuum conditions at (b) 700 °C for 40 min and (c) 750 °C for 80 min.

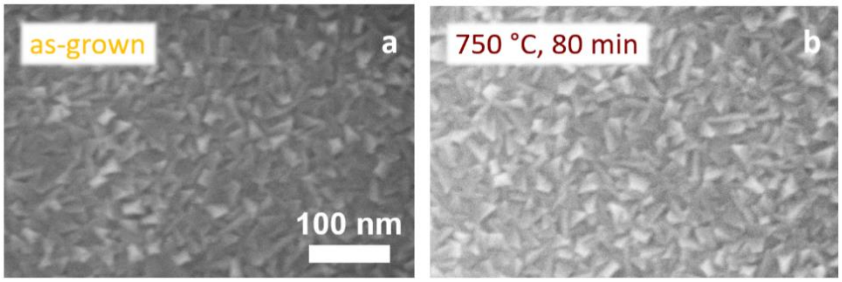

Figure S9. Scanning electron microscopy (SEM) images of a sample in (a) the as-grown state and (b) after annealing in vacuum at 750 °C for 80 min. The scale bar applies to both images.

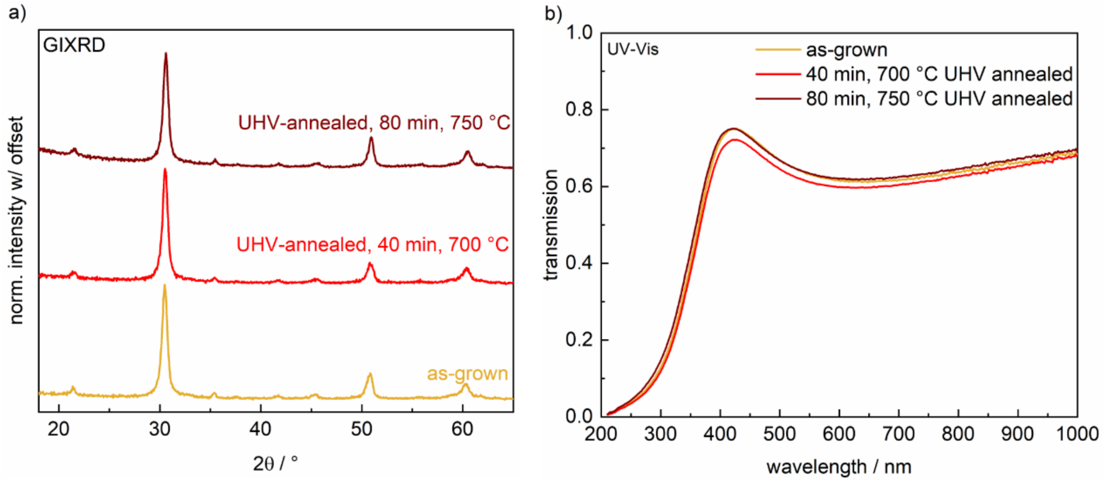

Figure S10. (a) Grazing incidence X-ray diffraction (GIXRD) patterns of as-grown and UHV-annealed (40 min at 700 °C and 80 min at 750 °C) Zr_2ON_2 thin films. (b) UV-vis transmission measurements of the same films shown in (a).

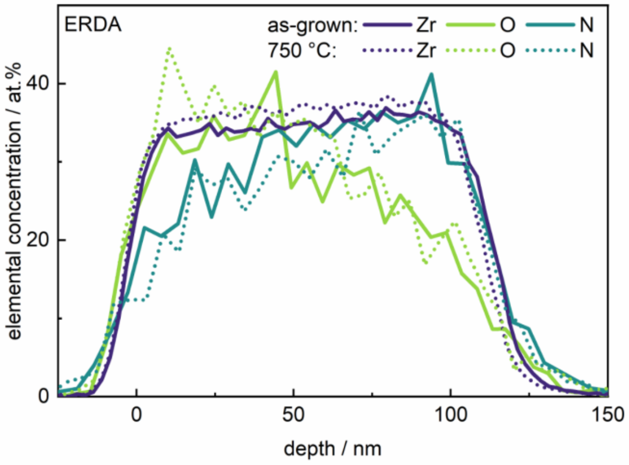

Figure S11. Elastic recoil detection analysis (ERDA) of the composition profiles within Zr_2ON_2 samples, comparing the as-grown film with one subsequently annealed at 750 °C for 80 min under high-vacuum conditions. Post-synthetic annealing leads to oxygen enrichment in the surface-near region, but the bulk remains unchanged, retaining the oxygen and nitrogen concentration gradients described in the main text.

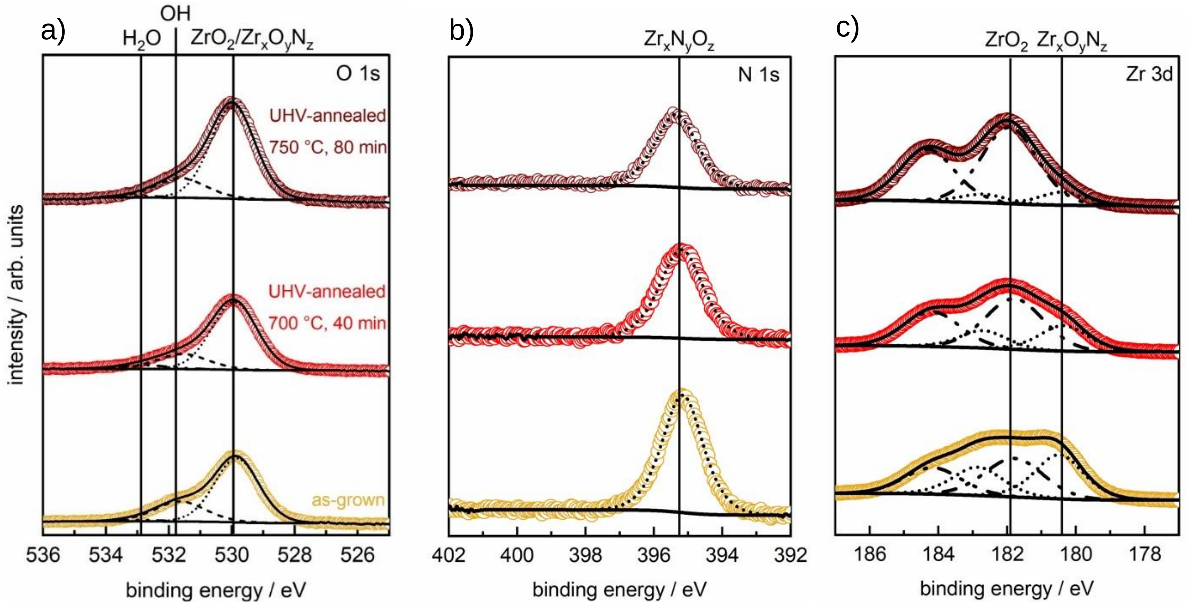

Figure S12. XPS core level spectra of the as-grown and UHV-annealed films shown in Figure S10. Annealing leads to a surface enrichment with oxygen and a depletion of nitrogen, as shown by both increasing (a) O 1s and decreasing (b) N 1s intensities, as well as by a decreasing fraction of the $Zr_xO_yN_z$ contribution compared to the ZrO_2 contribution in the (c) Zr 3d spectrum.

Supplemental Table

Table S1. Indirect and direct band gaps of the sample series, as determined by Tauc analysis.

Sample name	Indirect band gap / eV	Direct band gap / eV
0 sccm O_2	2.7	3.2
0.08 sccm O_2	2.9	3.3
0.12 sccm O_2	3.1	3.6
0.16 sccm O_2	3.3	3.8
0.20 sccm O_2	3.4	4.0
0.24 sccm O_2	3.4	4.1
0.28 sccm O_2	3.6	4.3
0.32 sccm O_2	3.8	4.5
